# AutoGP: An intelligent breeding platform for enhancing maize genomic selection

**DOI:** 10.1016/j.xplc.2025.101240

**Published:** 2025-01-08

**Authors:** Hao Wu, Rui Han, Liang Zhao, Mengyao Liu, Hong Chen, Weifu Li, Lin Li

**Affiliations:** 1 College of Informatics, Huazhong Agricultural University, Wuhan 430070, China; 2 Engineering Research Center of Intelligent Technology for Agriculture, Ministry of Education, Wuhan, China; 3 National Key Laboratory of Crop Genetic Improvement, Huazhong Agricultural University, Wuhan 430070, China; 4 Hubei Hongshan Laboratory, Hubei, China

**Keywords:** smart breeding, genomic selection, phenotypic extraction, deep learning, maize, breeding platform

## Abstract

In the face of climate change and the growing global population, there is an urgent need to accelerate the development of high-yielding crop varieties. To this end, vast amounts of genotype-to-phenotype data have been collected, and many machine learning (ML) models have been developed to predict phenotype from a given genotype. However, the requirement for high densities of single-nucleotide polymorphisms (SNPs) and the labor-intensive collection of phenotypic data are hampering the use of these models to advance breeding. Furthermore, recently developed genomic selection (GS) models, such as deep learning (DL), are complicated and inconvenient for breeders to navigate and optimize within their breeding programs. Here, we present the development of an intelligent breeding platform named AutoGP (http://autogp.hzau.edu.cn), which integrates genotype extraction, phenotypic extraction, and GS models of genotype-to-phenotype data within a user-friendly web interface. AutoGP has three main advantages over previously developed platforms: 1) an efficient sequencing chip to identify high-quality, high-confidence SNPs throughout gene-regulatory networks; 2) a complete workflow for extraction of plant phenotypes (such as plant height and leaf count) from smartphone-captured video; and 3) a broad model pool, enabling users to select from five ML models (support vector machine, extreme gradient boosting, gradient-boosted decision tree, multilayer perceptron, and random forest) and four commonly used DL models (deep learning genomic selection, deep learning genomic-wide association study, deep neural network for genomic prediction, and SoyDNGP). For the convenience of breeders, we use datasets from the maize (*Zea mays*) complete-diallel design plus unbalanced breeding-like inter-cross population as a case study to demonstrate the usefulness of AutoGP. We show that our genotype chips can effectively extract high-quality SNPs associated with days to tasseling and plant height. The models show reliable predictive accuracy on different populations and can provide effective guidance for breeders. Overall, AutoGP offers a practical solution to streamline the breeding process, enabling breeders to achieve more efficient and accurate genomic selection.

## Teaser

This study introduces AutoGP, an intelligent breeding platform designed to enhance maize genomic selection through an integrated and user-friendly web interface. AutoGP combines efficient genotype and phenotypic extraction workflows with advanced genomic selection models, offering breeders access to a diverse pool of machine learning and deep learning tools. By demonstrating reliable predictive accuracy using the maize CUBIC population, AutoGP provides practical solutions to streamline breeding processes and accelerate crop improvement.

## Introduction

Due to challenges from an increasing population and climate change, there is an urgent need to cultivate elite germplasm that is both high yielding and resistant to multiple biotic and abiotic stresses (Liu et al., 2024). The advent of genomic selection (GS)-aided breeding technologies has provided an unprecedented opportunity to rapidly and efficiently develop new varieties to promote global food security (Zhu et al., 2024).

As a transformative force in plant breeding, GS promises to accelerate genetic gain and address the growing demand for agricultural products. The concept of GS was first introduced in livestock breeding, driven by the need for faster genetic improvement (Meuwissen et al., 2001). Various statistical algorithms have been developed and applied to leverage the power of genomic data, forming the core of GS. Two prominent algorithms, genomic best linear unbiased prediction and ridge regression best linear unbiased prediction (rrBLUP), are widely used in GS (Wang et al., 2015; Xu et al., 2018). With the advent of biological big-data techniques, several advanced GS methods with improved predictive accuracy incorporating multiomics data have been developed; notably, the rise of AI-based methods has further advanced GS. Accordingly, machine learning (ML) methods (support vector machine [SVM], extreme gradient boosting [XGBoost], gradient-boosted decision tree [GBDT], multilayer perceptron [MLP], and random forest [RF]) and deep learning (DL) methods (deep learning genomic selection [DeepGS] [Ma et al., 2018], deep learning genomic-wide association study [DLGWAS] [Liu et al., 2019], deep neural network for genomic prediction [DNNGP] [Wang et al., 2023], and SoyDNGP [Gao et al., 2023]) have been developed as powerful GS methods. However, these methods have not been widely used by breeders. They are more complex and costly, presenting a significant challenge for breeders to navigate and optimize within their breeding programs.

**Figure 1:**
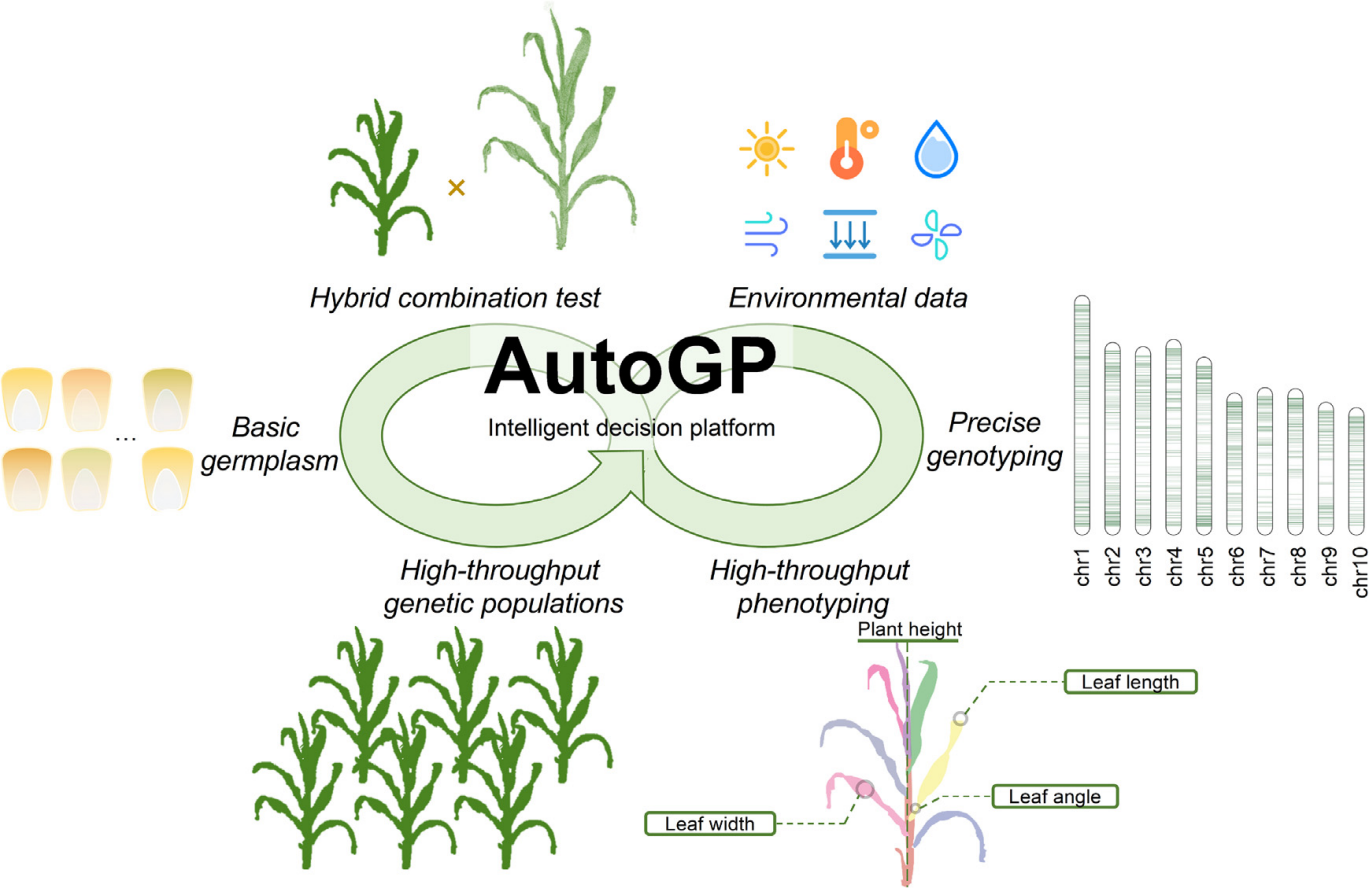
AutoGP, a new intelligent breeding framework. Breeders can create large-scale populations using selfing or hybridization techniques with existing seed stocks. Genotyping data can be efficiently obtained by advanced chip sequencing. Separately, 3D point cloud algorithms enable the precise monitoring of crop phenotypes. By combining these data, the platform trains models with high predictive accuracy. AutoGP not only predicts phenotypes for unknown samples but also guides breeders in selecting the best parental combinations.

Here, we present an intelligent breeding framework (Figure 1) called AutoGP, with the aim of bridging the gap between the sophisticated technical expertise typically required for GS and the practical needs of breeders. AutoGP automatically integrates a genotyping system with a precision design genotyping array of ∼5000 functional genes, a light and hand-held field phenotyping system, and a bioinformatics pipeline containing all released GS models. Breeders can execute all operations associated with GS within their breeding programs through a single integrated interface, including the collection and analysis of genotype and phenotype data, the construction of GS models, and subsequent predictions. We demonstrate the simplicity and power of AutoGP for the prediction of plant height in maize (*Zea mays*) as a proof of concept. This platform is engineered to simplify access to GS methodologies, thereby enhancing their integration into breeding practices. AutoGP can be used for a wide range of species and dramatically advance the applications of GS in breeding.

## Results and discussion

The rapid accumulation of genotype-to-phenotype (G2P) data has laid a solid foundation for crop breeding. Effective management of genotypic, environmental, and phenotypic data is fundamental to GS research. We offer three types of databases in AutoGP—a personal database, a shared database (with unpublished data of uncertain quality), and a high-quality database (with published and validated data)—accommodating data uploading, querying, storage, and sharing. For reference, some of the many high-quality databases include a dataset of 20 087 soybean (*Glycine max*) materials with 42 509 high-confidence single-nucleotide polymorphisms (SNPs) (Grant et al., 2010), a wheat (*Triticum aestivum*) dataset of 2000 materials genotyped with 33 709 diversity array technology markers (Crossa et al., 2016), a maize dataset with 42 840 F_1_ hybrids generated from crosses between 1428 maternal and 30 paternal lines (Liu et al., 2020), multiple tomato (*Solanum lycopersicum*) datasets (Zhou et al., 2022), a comprehensive collection of over 224 billion genotype data points (individual SNPs) and 434 000 phenotypic data points (individual phenotype records for a single sample) generated from >30 000 individuals of 14 representative populations of 7 major crop species (Chen et al., 2024), and a dataset of 143 477 rice (*Oryza sativa*) G2P paired data for 41 traits, 284 395 soybean G2P paired data for 114 traits, and 12 654 maize G2P paired data for 18 traits (Shen et al., 2024).

With these assembled datasets, many platforms with AI-aided models have been proposed to accelerate the development of breeding technologies. Specifically, CropGS-Hub (Chen et al., 2024) provides six different ML models (BayesCpi, bayesian lasso, bayesian regression, genomic best linear unbiased prediction, ridge regression best linear unbiased prediction, and light gradient boosting machine). BreedingAIDB (Shen et al., 2024) provides the model with three different boosting types. The Smart Breeding Platform (Li et al., 2024) enables the user to perform GS using ML and DL models (Montesinos-López et al., 2021). Unlike these platforms, AutoGP integrates genotype chips, phenotype extraction, and a GS model pool into a user-friendly web interface. Specifically, we are the first to design an efficient sequencing chip that identifies high-quality, high-confidence SNPs across gene-regulatory networks and the first to develop a complete workflow for extraction of plant phenotypes, such as plant height and leaf count, from smartphone-captured videos. In addition, we offer a comprehensive model pool consisting of five ML models and four widely used DL models, surpassing the range available on other platforms.

### Extraction of high-quality SNPs from gene-regulatory networks

**Figure 2:**
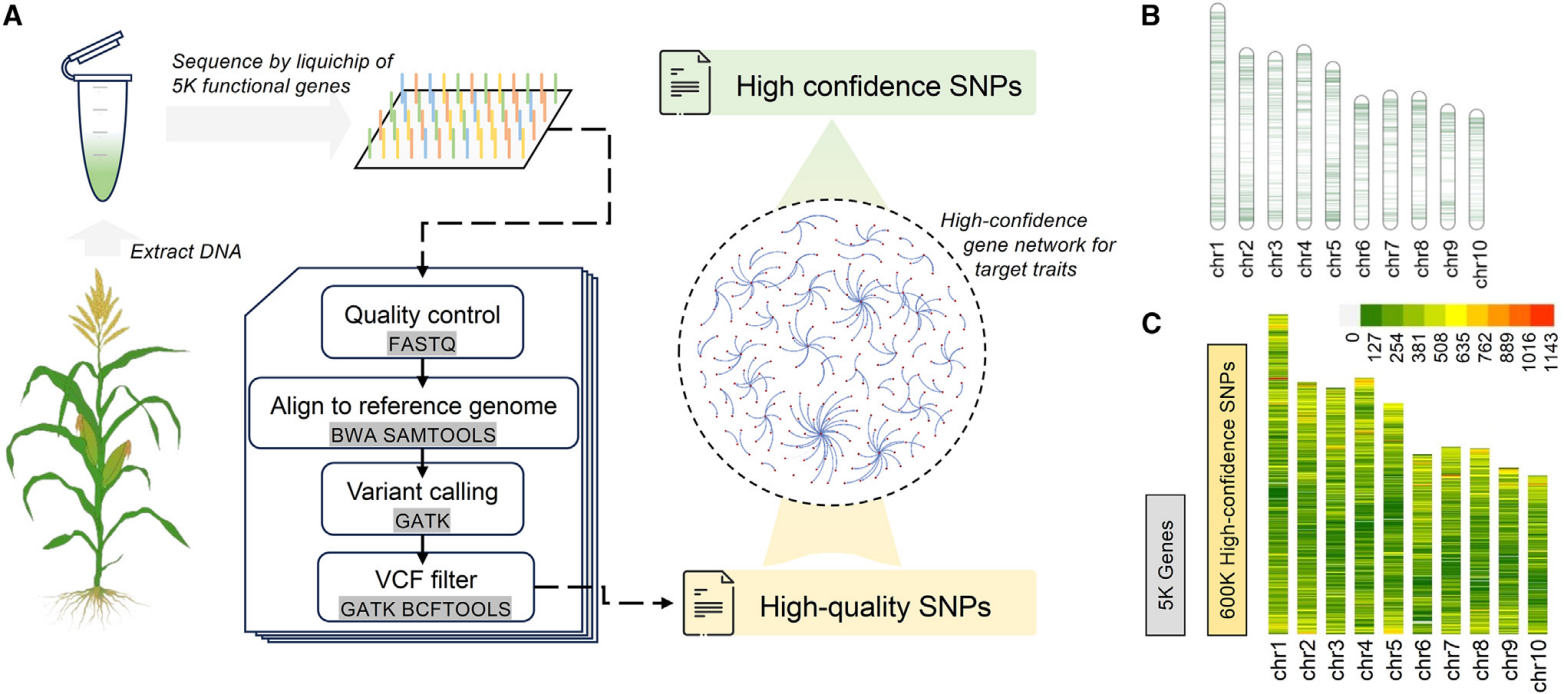
High-confidence genotyping platform for targeted functional genes. **(A)** High-throughput sequencing of 5061 target genes was performed by sequencing genomic DNA extracted from target plants using 5K liquid-phase gene-chip microarrays. The information on high-quality SNPs is obtained through variant detection in the sequencing data, and key SNPs are extracted on the basis of high-confidence gene interaction networks for the target traits. By obtaining variant loci that are more relevant to the target phenotype, it is possible to achieve crop genetic improvement with precise genomic trait selection programs. **(B)** Distribution of the 5061 genes targeted by gene-chip sequencing along the maize chromosomes. **(C)** Distribution of 600 000 (600K) high-confidence SNPs on chromosomes within the 5061 gene regions and their upstream and downstream regulatory regions (2 kb upstream, 1 kb downstream).

GS leverages the genetic diversity embedded within genomes; however, the construction of models with high-density molecular markers across the entire genome is often prohibitively expensive owing to the high cost of sequencing. However, models based on low-density markers tend to yield suboptimal results. In modern breeding, specific candidate genes, rather than the entire genome, are the primary interest. To address this limitation, a comprehensive gene-regulatory network was previously developed for maize, encompassing high-confidence functional genes associated with key traits such as days to tasseling (DTT) and plant height (PH) (Han et al., 2023). This approach used liquid-phase microarray sequencing of 5061 functional genes (Figure 2B), facilitating the identification of high-quality SNPs linked to the critical traits associated with these genes. Because genes do not function as isolated units but instead interact within complex networks, the marginal effects of individual genes can vary under different environmental conditions; we therefore advocate for extraction of high-confidence SNP features associated with the target traits (DTT and PH) within the context of their gene-regulatory networks. AutoGP enables the convenient utilization of these high-quality SNPs selected from gene interaction networks. Because their explanatory power for the target traits is stronger than that of more conventional methods, it is possible to obtain more cost-effective and efficient genomic predictions with fewer genetic features and higher confidence.

### Complete workflow for phenotype extraction

**Figure 3:**
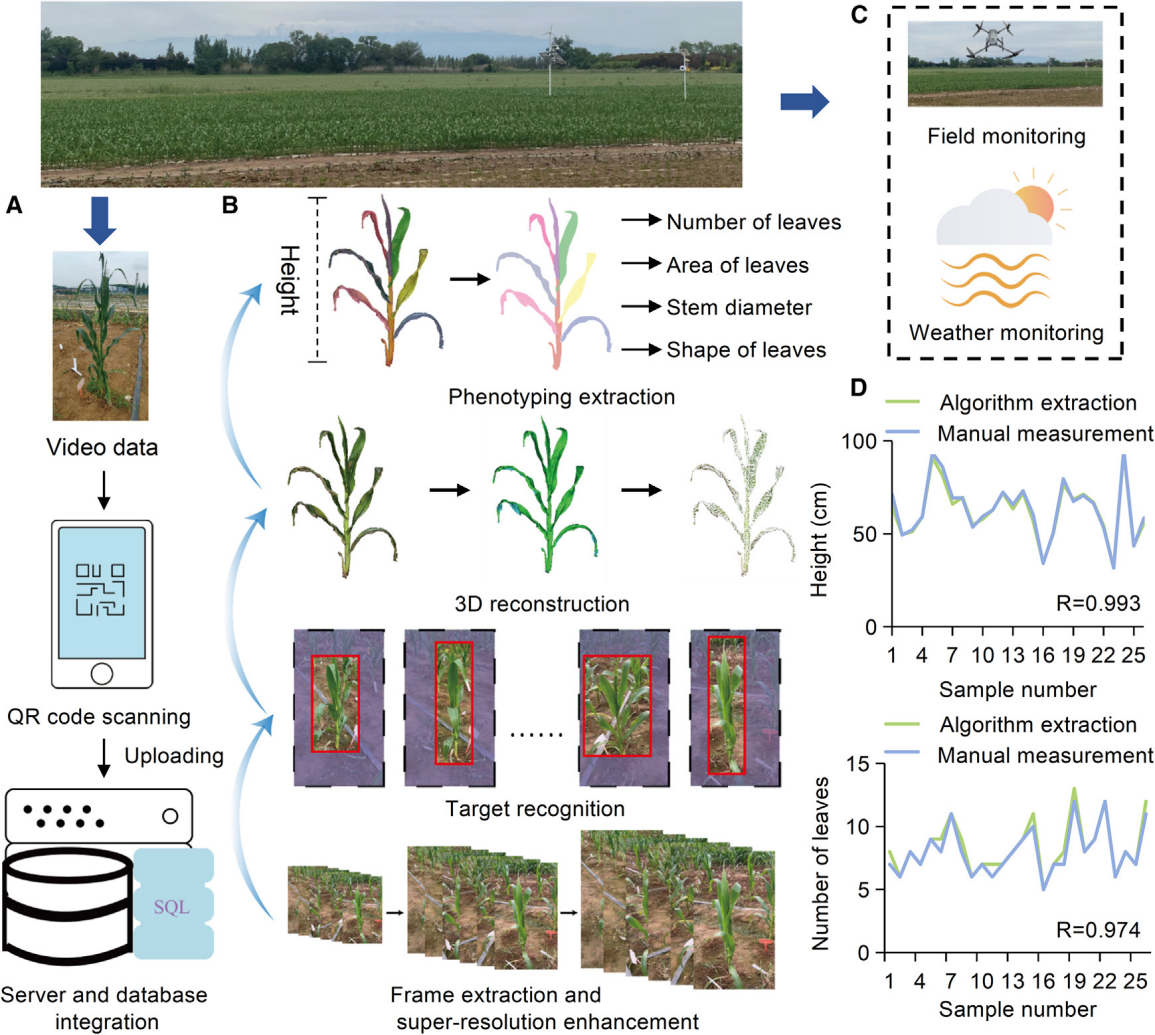
Acquisition of phenotypic data and phenotyping. **(A)** Video data collection. A QR code displayed in the “Phenotype extraction” module of AutoGP can be scanned with a smartphone to open a WeChat mini-program, enabling users to record videos of individual plants and upload the footage to the server. **(B)** Plant phenotyping. The server processes incoming videos by extracting frames, using object recognition to remove the background, and performing 3D reconstruction to segment and quantify traits such as leaf area and leaf count. **(C)** Field phenotyping. An unmanned aerial vehicle captures aerial photographs to obtain hyperspectral data for traits such as chlorophyll level, and local meteorological stations record weather information. **(D)** Preliminary verification of PH and leaf count by comparing the values extracted by the algorithm to those collected manually. The mean absolute errors for PH and leaf count were 1.527 cm and 0.308, respectively, and the Pearson correlation coefficients (*R*) exceeded 0.95.

Traditional manual phenotyping is typically time consuming, irreproducible, labor intensive, and error prone, making high-throughput phenotyping techniques necessary for multiomics analyses and the systematic investigation of plant gene functions (Li et al., 2022b; Ma et al., 2023; Sun et al., 2024; Zhang et al., 2024). To reduce the amount of manual labor required for high-throughput phenotype extraction (Ma et al., 2021; Guo et al., 2023), we developed a complete workflow using a WeChat mini-program for data collection and advanced 3D Gaussian splatting for 3D reconstruction, which are followed by a point cloud segmentation algorithm or web-based interactive segmentation method for phenotype extraction (Li et al., 2021; Xu et al., 2022; Zhou et al., 2023). With just a smartphone, users can obtain detailed 3D plant models and comprehensive data for phenotypes such as PH and total leaf number, which are essential for agricultural research and breeding programs (Figure 3A and 3B). Meteorological data can be collected from a nearby station (Figure 3C). The proposed workflow not only minimizes the manual labor typically associated with high-throughput phenotype extraction but also makes sophisticated phenotyping tools available to all. Researchers can benefit from accurate, rapid, and cost-effective phenotype analysis.

To assess the effectiveness of AutoGP, mobile phones were used to capture 26 videos of maize in the field, and PH and leaf count were quantified by a human researcher. The phenotype values extracted using our workflow were very close to those collected by humans, with Pearson correlation coefficients (*R*) exceeding 0.95. The mean absolute error for PH and leaf count were 1.527 cm and 0.308, respectively, validating the accuracy of phenotype extraction (Figure 3D). Given the complexities of the field environment, the 3D Gaussian splatting and point cloud segmentation algorithm might fail or become ineffective. For this reason, we also provide an interactive interface, enabling researchers to easily modify the identified result through simple clicks. Precise phenotypes, such as leaf length and width, leaf area, and leaf angle, can also be extracted using this approach.

### Complete pipeline for genomic selection

**Figure 4:**
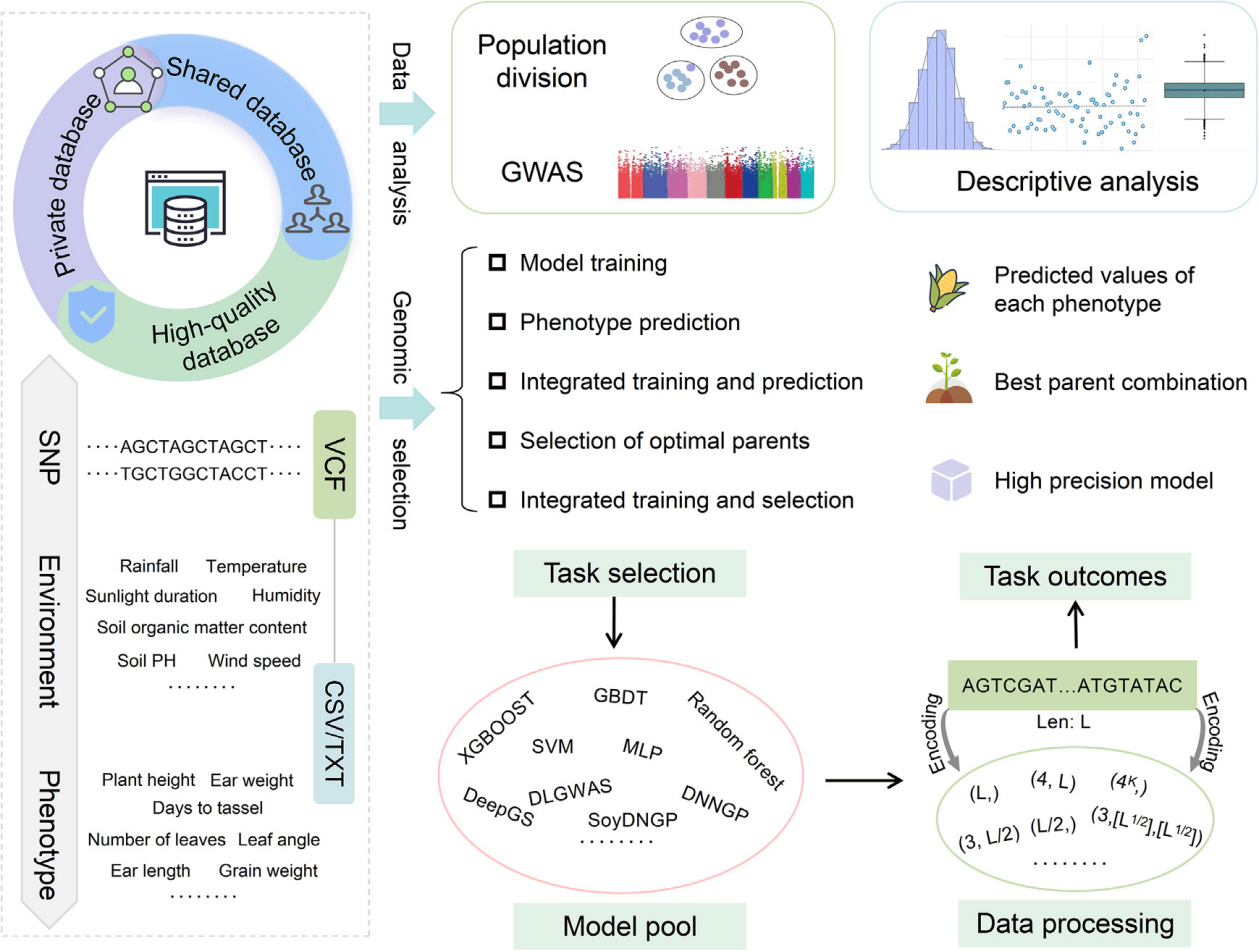
Data management and genomic selection strategies. The “Data management” module ensures data security, privacy, and quality, offering private, shared, and high-quality databases. The “Data analysis” module enables in-depth analysis of VCF data, performs a simple statistical characterization of phenotypic data, and includes intuitive visualization tools. The “Genome selection” module integrates multiple algorithms for model training, phenotype prediction, training–prediction integration, selection of optimal parents, and training–selection integration.

We allow users to select a single model from a model pool containing five ML models (SVM, XGBoost, GBDT, MLP, and RF) and four commonly used DL models (DeepGS, DLGWAS, DNNGP, and SoyDNGP), providing flexibility to help users identify the optimal model for their system. AutoGP provides five functional modules for GS (Figure 4): model training, phenotype prediction, integrated training and prediction, selection of optimal parents, and integrated training and selection. The corresponding inputs, outputs, and implementation are detailed below.

#### Model training

AutoGP provides a model pool for user selection on the basis of the breeding program. The input for training includes genotype data (variant call format [VCF] file) and corresponding phenotype data (comma separated values [CSV] file). The output is a model weight file together with a predictive accuracy (*R* value), which can be used for subsequent phenotype prediction and transfer learning.

#### Phenotype prediction

AutoGP enables users to predict a target phenotype from available genotype data by submitting genotype data (VCF file) and a model weight file from the “Model training” module above. Within minutes, a CSV file with the predicted phenotypes is generated. The predictive accuracy depends on the predictive capability of the selected model and the differences between the prediction and training populations. To ensure reliability, users should select high-accuracy model weights and similar populations.

#### Integrated training and prediction

To streamline the procedure for users, AutoGP integrates the “Model training” and “Phenotype prediction” functionalities into a unified module. This integration ensures a seamless experience in which users can train models and predict phenotypes in a single step.

#### Selection of optimal parents

AutoGP enables users to predict the F_1_ phenotypes of various hybrid combinations, facilitating the optimal selection of parents. Users provide genotype data from inbred lines of the crop of interest (text file), VCF data of potential parental combinations, and a model weight file from the “Model training” module. The output includes a CSV file with the predicted F_1_ phenotypes and provides the top five parental combinations that match the desired traits.

#### Integrated training and selection

Integration of model training with selection of optimal parents streamlines the procedure for users. Users input genotype (VCF) and phenotype (CSV) data to train a predictive model, resulting in a model weight file with a high accuracy (*R* value). By submitting genotype data from inbred lines of the crop of interest (text file) and VCF data on potential parental combinations, the generated model file predicts F_1_ phenotypes and identifies the top five parental combinations that best match the target traits.

Furthermore, AutoGP offers dedicated environmental information-embedded modules for model training, phenotype prediction, and integrated training and prediction. This enables users to use a single model for phenotype prediction across different regions. Note that phenotypic variation is associated with the complex interplay between genomic sequences, transcript levels, and environmental parameters; thus, integration of environmental factors and various omics data improves the precision of GS models (Xu et al., 2021). In future work, the AutoGP platform will focus on predicting phenotypic traits from multimodal data (Li et al., 2022a).

### A case study on the CUBIC population

The large-scale dataset for the CUBIC population was collected in 2014 and 2015 (Yan et al., 2021). The NCII6210 dataset, which is the portion of the dataset with paired genotype and phenotype data, consists of 6210 F_1_ hybrids (207 female × 30 male) collected across 5 provinces in China (Liaoning, Jilin, Beijing, Hebei, and Henan). The genotype data include 4 549 838 imputed SNPs based on the maize B73_v3.25 reference genome. We used the DTT and PH phenotypes as a case study.

#### Extraction of high-quality SNPs

High-confidence SNPs for the two traits (DTT and PH) were screened by looking at the overlap between SNPs and high-confidence functional gene regions and their corresponding regulatory areas. As a result, we identified 14 075 SNPs related to DTT and 20 738 SNPs related to PH (Figure 2C).

#### Performance of GS models

**Figure 5:**
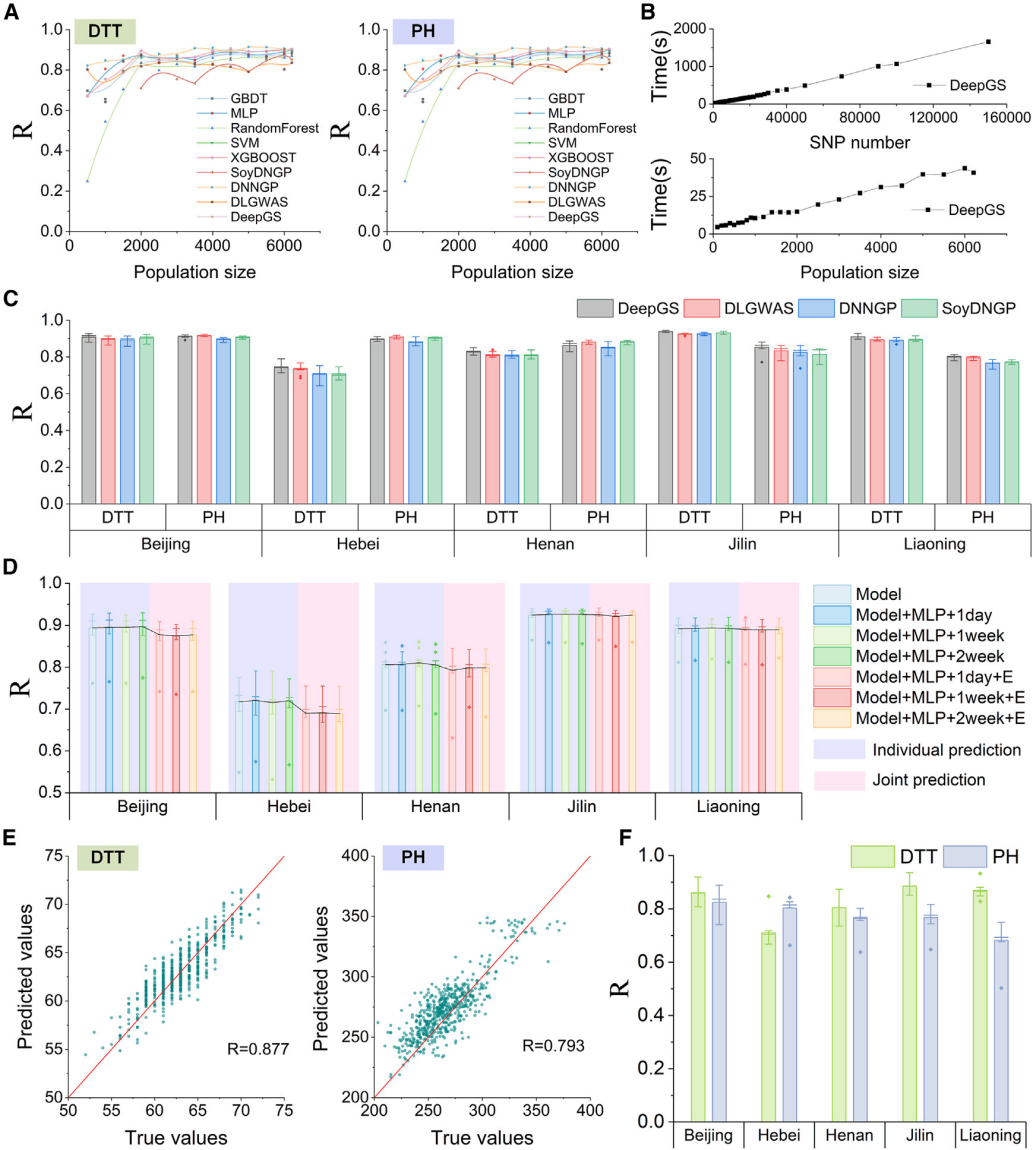
Performance and application of AutoGP on the NCII6210 dataset. **(A)** The prediction accuracy *R* of days to tasseling (DTT) and plant height (PH) of various GS models using different population sizes, which were randomly sampled from the NCII6210 dataset. **(B)** The runtime of the DeepGS model in a single run is shown as a function of SNP number for 6210 individuals (top) and as a function of population size (bottom), where SNPs were randomly selected from the entire genome. **(C)** Prediction accuracy of four DL methods for maize phenotyping across five different provinces. For each province, a separate model was trained for each phenotype. **(D)** Comparison of DTT predictions using a simple CNN model alone versus a CNN model enhanced with an MLP that incorporates environmental data (daily, weekly, and biweekly) for individual provinces. The notation “+daily, weekly, or biweekly” indicates that the model embeds the corresponding time-series environmental information, and “+E” signifies that a single model is trained on multiple environmental datasets simultaneously. **(E)** Scatterplots of the collected DTT and PH data (x axes) and the predicted results (y axes). **(F)***R* values for different provinces. All of the above results were obtained using a single A6000 graphics processing unit.

We investigated several GS models using different population sizes (500–6210) selected from the NCII6210 dataset. The prediction accuracy across various population sizes is presented in Figure 5A. The *R* value of all GS models exceeded 0.75 for DTT and 0.8 for PH when the population size reached 2000 and above. Beyond this point, further increases in population size yielded only marginal improvements. We also examined the time required to complete one DL model with different populations and different numbers of SNPs (1000–160 000) (Figure 5B). The running time increased with both factors. We used four DL methods for GS training on the NCII6210 dataset, with consistently good AutoGP predictive performance across multiple regions through repeated experiments (Figure 5C).

#### Environmental information-embedded GS model

As the environmental information for the NCII6210 dataset, we extracted 11 key environmental variables from Visual Crossing: maximum temperature, minimum temperature, humidity, precipitation, wind speed, sea level pressure, cloud cover, solar radiation, solar energy, UV index, and sunlight duration. Because the abovementioned GS methods are not designed to incorporate environmental information, we referred to the squeeze-and-excitation network (Hu et al., 2018), which uses a convolutional neural network (CNN) to encode the genotype data and an MLP to encode the environmental data into a 64-dimensional vector. The vector was used to reweight feature maps within the CNN, thereby effectively integrating environmental information. This design enables the model to predict phenotypes across different environments using a single framework. As shown in Figure 5D, although the combination of a simple CNN and MLP leads to a slight decrease in predictive accuracy across multiple provinces, the model still maintains substantial predictive effectiveness.

#### Guidance for breeders

To simulate real-world breeding, we divided the NCII6210 dataset into 5610 hybrid samples (crosses between 187 female and 30 male inbred lines) for training and another set of 600 hybrid samples (crosses between 20 female and 30 male inbred lines) for analysis as candidates. We used the training data to obtain environment-specific weight files using the functional module “Model training.” The functional module “Phenotype prediction” showed that the *R* values between predicted and manually collected DTT and PH data exceeded 0.7 across the five provinces (Figure 5E and 5F). This result demonstrates the significant potential of using AutoGP to guide accurate prediction of hybrid offspring phenotypes. Taking PH as an example, the functional module “Selection of optimal parents” predicted 5 hybrid combinations with a higher PH for each province from the list of 600 candidates. The single top-performing hybrid of the predicted 5 hybrids in each province ranked 13th, 2nd, 1st, 3rd, and 2nd of 600. This finding demonstrates the reliability of AutoGP in guiding the selection of superior hybrids, thus improving breeding efficiency.

### Limitations of AutoGP

AutoGP is an intelligent breeding platform designed to facilitate extraction of genotype and phenotype data, as well as G2P modeling, through a user-friendly web interface. It incorporates an efficient sequencing chip that identifies high-quality SNPs within gene-regulatory networks, alongside a comprehensive workflow for extraction of plant phenotypes from smartphone-captured videos. In addition, AutoGP provides a diverse array of ML and DL models for user selection. Despite its advances, AutoGP has several limitations in its application to breeding. First, when using AutoGP, users must preprocess their data into formats compatible with the platform, such as specific VCF formats for genotype data and CSV formats for phenotype data. Second, AutoGP cannot provide high-quality SNPs for target phenotypes that lack associated regulatory networks, necessitating reliance on whole-genome data or feature-selected data for further research. In addition, it takes several minutes for the backend to model each video for phenotype extraction and to train the GS models. Lastly, the default set of hyperparameters may not yield optimal GS models. Future work will focus on improving capabilities by supporting a wider range of data formats, expanding gene-regulatory networks for different phenotypes, enhancing phenotype extraction speed, and implementing automatic hyperparameter tuning or enabling user-driven adjustments.

## Methods

### Acquisition of high-confidence SNPs

To obtain high-confidence SNPs associated with particular phenotypes from any target population, the program presented in Figure 2A can be followed. Genomic DNA is extracted from an individual in the target population, followed by sequencing of 5061 functional gene regions using a 5K liquid-phase microarray, providing comprehensive microarray sequencing data. During variant detection, the GenomicsDBImport tool from GATK4 is used to construct a genomic variant call format (gVCF) database. This method allows for future addition or removal of samples by direct modification of the gVCF files in the database, enhancing scalability and eliminating the need to use the CombineGVCFs function from GATK3, which requires a recalculation with all gVCF files.

After initial variant detection, the SNP variants must be selected and filtered for quality (QD < 2.0, QUAL < 30.0, FS > 60.0, MQ < 40.0, SOR > 3.0, MQRankSum < -12.5, MQRankSum_low_-12.5), loci with a 95% missing rate must be removed, and genotypes of the detected variants must be imputed when data are missing. At this point, high-quality SNP data are obtained. These SNPs are further refined by leveraging functional gene interactions and regulatory networks specific to the target traits. A 3-kb regulatory region is defined around each gene by expanding the sequence 2 kb upstream and 1 kb downstream from the coding sequence of each functional gene subjected to chip sequencing. The SNPs within these expanded 3-kb regions are included in the GS models as regulatory region features. The coding sequence intervals of the functional genes and their 3-kb regulatory regions are cross-referenced with the loci in the VCF file. This step identifies key features in the VCF data that correspond to the high-confidence functional gene interaction regulatory networks for the target traits, ensuring precise and reliable selection of SNPs for GS.

For public datasets and historically stored data, this analysis procedure can still be used to achieve a relatively accurate GS prediction. For sequencing data stored in FASTQ format, it is necessary to first obtain a large number of SNP variant features through the variant detection analysis pipeline (Figure 2A). Subsequently, key SNP features can be extracted through the functional gene interaction network corresponding to the target traits. Although many SNPs can be obtained from previous sequencing datasets, their coverage of gene regions in the target functional gene interaction network may be limited compared with the higher specificity provided by the 5K liquid-phase microarray data generated with the gene chips. This discrepancy can lead to decreased prediction accuracy. In VCF datasets that have been subjected to variant calling, the number of variants that intersect with the regions targeted by the 5K liquid-phase microarray is typically lower compared with the number of variants that can be extracted directly from the raw sequencing data in FASTQ format owing to the distinct research objectives. In such cases, only SNP features that overlap with the functional gene intervals of the target traits can be identified as high-confidence SNPs.

### Phenotype extraction

A simple video collection strategy was developed to reduce the manual labor required for high-throughput phenotype extraction. Users can employ smartphone cameras to perform 360° panoramic imaging of targeted plants and then upload the video via a WeChat mini-program. During data collection, the plant variety and the photographer’s name are recorded to ensure data accuracy and traceability. Users have the flexibility to either capture videos on-site in real time or upload pre-recorded videos from their local storage. To accommodate the on-site network environment, the video is automatically compressed before uploading (Hu et al., 2024).

During 3D reconstruction of the phenotypic data, frame extraction processing is performed on the uploaded video. The image quality is then enhanced through super-resolution processing with RealBasicVSR (Chan et al., 2022). Finally, an advanced 3D reconstruction algorithm is applied to refine the analysis (Mildenhall et al., 2021; Kerbl et al., 2023). Once the reconstruction is complete, users receive feedback on the success of the data processing and the quality of the reconstructed 3D model, which is then stored on AutoGP (Figure 3A and 3B).

The 3D model is first converted into a point cloud, and the density-based spatial clustering of applications with noise clustering algorithm is applied for noise reduction. Principal-component analysis (PCA) is used to correct axis deviations, and central cropping is performed to remove field noise. Point cloud segmentation algorithms or web-based interactions are then used to distinguish various plant parts, such as leaves and stems (Adams et al., 2020; Shoaib et al., 2022). Specifically, PH is determined by calculating the maximum difference in the vertical coordinates of the stem point cloud, and the number of leaves is obtained by instance labeling.

### Data management

Effective management of genotypic, environmental, and phenotypic data is fundamental to GS research. AutoGP provides robust solutions for data management (Figure 4). AutoGP offers personal databases that ensure privacy, shared databases that promote collaboration, and high-quality databases curated by platform professionals that provide reliable, rigorously selected data for GS research.

Users of AutoGP can submit data in various modules by two methods: local data upload and selection of database data. Both methods guarantee the privacy of user data. When users opt for local data upload, the platform ensures that all uploaded source data are completely deleted upon completion of the target task. When database data are selected, personal databases are accessible only to the individual user.

### ML and DL algorithms for GS modeling

#### ML methods

RF constructs multiple decision trees by randomly selecting subsets of samples and features from the original data for training. Each tree determines the optimal split using metrics such as the Gini index to minimize the loss function. RF combines and averages the outputs of all decision trees. SVM constructs hyperplanes to maximize the margin between different classes for classification or regression tasks. It uses kernel functions to map data from a low-dimensional to a high-dimensional space, enabling it to capture nonlinear relationships that are not easily separable in the original space. GBDTs enhance model performance by constructing multiple weak learners, typically decision trees. Each new decision tree is trained on the residuals of the previous model to iteratively decrease prediction errors. As an improved version of GBDT, XGBoost incorporates regularization terms to prevent overfitting and employs more efficient computation methods to enhance training speed and model performance. MLP is a feedforward neural network that transforms input data through multiple layers of fully connected neurons. This procedure incrementally extracts features and performs nonlinear mappings. The output of each layer serves as the input for the next, with the final output providing the prediction result.

The DeepGS model (Ma et al., 2018), based on CNNs, demonstrates exceptional performance in prediction of grain-related phenotypes in wheat populations. Its fundamental architecture comprises a convolutional layer, a pooling layer, and a fully connected layer followed by a rectified linear unit activation function. To prevent overfitting, a dropout rate of 0.05 is applied. This model architecture is simple yet efficient, offering superior predictive capabilities.

DLGWAS (Liu et al., 2019) uses multiple parallel CNNs for phenotype prediction, demonstrating superior predictive performance compared with a single CNN model. DLGWAS processes raw data using convolutional layers at two different scales. These outputs are then combined through an add-up layer, followed by fully connected layers to predict the phenotype.

DNNGP (Wang et al., 2023) uses a multilayer CNN architecture with a dimensionality reduction of genotype data via a PCA. The resulting low-dimensional data are then processed through three convolutional layers and fully connected layers to predict the phenotype. A dropout layer is also applied during training to prevent overfitting.

SoyDNGP (Gao et al., 2023) incorporates design principles derived from the visual geometry group network (Simonyan and Zisserman, 2014). SoyDNGP is a low-parameter, high-accuracy approach with robust performance across various crops, including soybean, cotton (*Gossypium hirsutum*), maize, rice, and tomato. The core technique involves reshaping the genotype data into a matrix, after which the classical visual geometry group network is used to predict the phenotype.

### Training details of the GS models

The training of models on the AutoGP platform encompasses several critical steps, beginning with input data reception and algorithm selection. Users provide genotype and phenotype data along with their chosen GS model. For the ML methods and DeepGS, the genotype data are encoded into an L-dimensional vector for each crop. DLGWAS uses one-hot encoding to generate a (4, L) matrix, whereas SoyDNGP uses one-hot encoding and reshaping to create a (3, [L^1/2^], [L^1/2^]) tensor, where “[]” denotes rounding down. DNNGP leverages PCA for dimensionality reduction, encoding the data into a 150-dimensional vector. Subsequently, the encoded genotype tensor and corresponding phenotype are partitioned into training, validation, and test sets in an 8:1:1 ratio. Model training is performed using the adaptive moment estimation optimization algorithm with a batch size of 64. For regression tasks, the mean squared error is used as the loss function, and the model performance is evaluated using *R*.

## Data and code availability

All datasets used in this study are openly accessible to ensure transparency and reproducibility. The CUBIC population data used in this study are available at https://ftp.cngb.org/pub/CNSA/data3/CNP0001565/zeamap/99_MaizegoResources/01_CUBIC_related/. The maize B73_v3.25 reference genome can be accessed at https://ftp.ensemblgenomes.ebi.ac.uk/pub/plants/release-25/fasta/zea_mays/dna/Zea_mays.AGPv3.25.dna.toplevel.fa.gz. In addition, environmental data for this research were obtained from Visual Crossing (https://www.visualcrossing.com/).

## Funding

This research was supported by Biological Breeding-National Science and Technology Major Project (2023ZD04076), the 10.13039/501100012166National Key Research and Development Program of China (2023YFF1000100), the 10.13039/501100001809National Natural Science Foundation of China (32321005 and 32261143463), the Fundamental Research Funds for the 10.13039/501100012429Central Universities of China (2662024XXPY001), and the Outstanding Youth Team Cultivation Project of Center Universities (2662023PY007).

## Acknowledgments

This research was supported by Biological Breeding-National Science and Technology Major Project (2023ZD04076), the 10.13039/501100012166National Key Research and Development Program of China (2023YFF1000100), the 10.13039/501100001809National Natural Science Foundation of China (32321005 and 32261143463), the 10.13039/501100012226Fundamental Research Funds for the Central Universities of China (2662024XXPY001), and the Outstanding Youth Team Cultivation Project of Center Universities (2662023PY007). No conflict of interest is declared.
